# NSAID Exposure and Risk of Alzheimer's Disease: An Updated Meta-Analysis From Cohort Studies

**DOI:** 10.3389/fnagi.2018.00083

**Published:** 2018-03-28

**Authors:** Caixia Zhang, Yan Wang, Dongyin Wang, Jidong Zhang, Fangfang Zhang

**Affiliations:** ^1^Second Department of Neurology, Xinxiang Central Hospital, Xinxiang, China; ^2^Department of Neurology, Third Affiliated Hospital of Xinxiang Medical University, Xinxiang, China; ^3^Huixian People's Hospital of Henan Province, Henan, China; ^4^Huixian Second People's Hospital of Henan Province, Henan, China

**Keywords:** Alzheimer's disease, NSAID, anti-inflammatory drug, aspirin, cohort study

## Abstract

**Background:** Initial observational studies and a systematic review published recently have suggested that non-steroidal anti-inflammatory drug (NSAID) use has the trend to be associated with reduced risk of Alzheimer's disease (AD), while results remain conflicting. Thus, we performed an updated meta-analysis to reevaluate the evidence on this association.

**Methods:** Data sources from PUBMED, Embase and Cochrane Library from inception through April 2017 were searched by two independent reviewers. Eligible cohort studies were selected according to predefined keywords. We did a meta-analysis of available study data using a random-effects model to calculate overall relative risks (RRs) for associations between NSAID exposure and AD risk.

**Results:** From 121 potentially relevant studies, 16 cohort studies including 236,022 participants, published between 1995 and 2016, were included in this systematic review. Meta-analysis demonstrated that current or former NSAID use was significantly associated with reduced risk of AD (RR, 0.81, 95% CI0.70 to 0.94) compared with those who did not use NSAIDs. This association existed in studies including all NSAID types, but not in aspirin (RR, 0.89, 95% CI 0.70 to 1.13), acetaminophen (RR, 0.87, 95% CI 0.40 to 1.91) or non-aspirin NSAID (RR, 0.84, 95% CI 0.58 to 1.23).

**Conclusions:** Current evidence suggests that NSAID exposure might be significantly associated with reduced risk of AD. However, further large-scale prospective studies are needed to reevaluate this association, especially the associations in individual NSAID type.

## Introduction

Non-steroidal anti-inflammatory drugs (NSAIDs), as one of the most widely prescribed medications, are mostly used for relief of pain or inflammatory conditions. Previous epidemiologic studies have indicated that NSAID use can offer a protective effect on the development of Alzheimer disease (AD) (Etminan et al., 2003; McGeer and McGeer, 2007; Vlad et al., 2008). This hypothesis is supported by multiple mechanisms that NSAIDs could have an impact on AD (McGeer et al., 1996; Etminan et al., 2003; Szekely et al., 2004). NSAIDs have been indicated to serve as the blockade of cyclooxygenase (COX), leading to the decrease in the levels of prostaglandins, prostacyclin, and thromboxanes which are important substances in AD pathogenesis (Kotilinek et al., 2008; Choi and Bosetti, 2009; Woodling and Andreasson, 2016). Moreover, NSAIDs are also involved in the modulation of amyloid precursorprotein (APP) processing, inhibiting the formation of fibrillary Aβ and stimulate the secretion of the non-amyloidogenic α-secretase form of soluble APP, leading to a reduction of amyloidogenic forms (Avramovich et al., 2002; Eriksen et al., 2003; Hirohata et al., 2005; Kukar and Golde, 2008).

Several epidemiologic studies showed that patients treated with NSAIDs had a decreased risk for developing AD (Breitner et al., 2009; Côté et al., 2012; Chang et al., 2016a,b). However, this evidence was only based on observational studies. Still other studies did not find this association (Ancelin et al., 2012; Wichmann et al., 2016). Besides, there lacked clinical trials to obtain direct evidence for the effect of NSAIDs on the risk of AD. Due to the conflicting results, we aimed to reevaluate the existing uncertainty regarding the effects of NSAID exposure on risk of AD by updating the systematic review and meta-analysis of cohort studies.

## Methods

### Literature search

We performed this meta-analysis based on the Preferred Reporting Items for Systematic Reviews and Meta-Analysis checklist (PRISMA). We systematically conducted a literature search of several major databases including Pubmed, Embase, and the Cochrane Library on 15th April 2017using the keywords and medical subject heading (Mesh) terms: (Alzheimer Disease OR Alzheimer^*^ OR dement^*^) AND (non-steroid anti-inflammatory agent^*^ OR NSAIDs OR aspirin^*^). A thorough manual search of all related references in all selected studies was also conducted. The database search strategies were provided in Supplementary Search Strategy. A search for unpublished literature was not performed.

### Study selection and eligibility criteria

Studies were included if they met the following inclusion criteria: (i) the study applied a cohort study design including population-based or community-based cohort studies; (ii) the study investigated the associations between NSAID use and risk of developing AD; (iii) the study reported the risk estimates such as RRs or HRs and their corresponding 95% CIs, or indirect data for the calculation of the risk estimates. We excluded studies that did not satisfy the inclusion criteria or published in non-English language. CZ and YW independently searched databases, screened, selected titles and abstracts and conducted full-text review. If two or more cohort studies had overlapping samples, only one study with the most recent or comprehensive data was retained.

### Data extraction

The data extraction was performed two independent authors (CZ and YW) and cross-checked by DW and JZ using a predefined standardized data form. The following study characteristics were collected: first author, publication year, study design, population origin, sample size, country of origin, NSAID type, NSAID duration, NSAID exposure ascertainment, AD ascertainment, risk estimates, follow-up period and adjusted variables. Quality of the primary studies was assessed using the Newcastle-Ottawa Scale for cohort studies accordingly (Stang, 2010). We did not contact the original authors for missing data. Adjusted risk estimates with the largest number of adjusted variables were selected if multiple risk estimates were provided in the same cohort.

### Statistical analysis

Pooled RRs or HRs and 95% CIs were calculated for the associations between NSAID exposure and the risk of AD using a random effects model (DerSimonian and Laird, 1986). We conducted an analysis to investigate the association between any NSAID exposure and the risk of AD. Moreover, we also performed subgroup analyses by NSAID type (aspirin vs. acetaminophen vs. NSAID not aspirin), study design, study setting, study region, sample size and data collection method to assess the impact of these factors on our results. We did not analyse the relationship between the dose or duration of exposure to NSAID and risk of AD due to unavailability of the data.

We conducted meta-analysis using Stata® version 14.0 (Stata Corp LP, College Station, Texas, USA). Inter-study heterogeneity was assessed by the Cochran Q statistic and quantified by the I^2^ statistic with an I^2^ more than 50% indicating significant heterogeneity (Higgins et al., 2003). Finally, besides visual inspection of funnel plot asymmetry, publication bias was also examined using Egger's regression and Begg's rank correlation tests with a *P*-value < 0.05 as an indication of publication bias (Begg and Mazumdar, 1994; Egger et al., 1997). Duval's non-parametric trim-and-fill method was also used to explore the potential influence of publication bias by estimating the number of missing studies that might exist in a meta-analysis (Duval and Tweedie, 2000).

### Results

**Figure 1:**
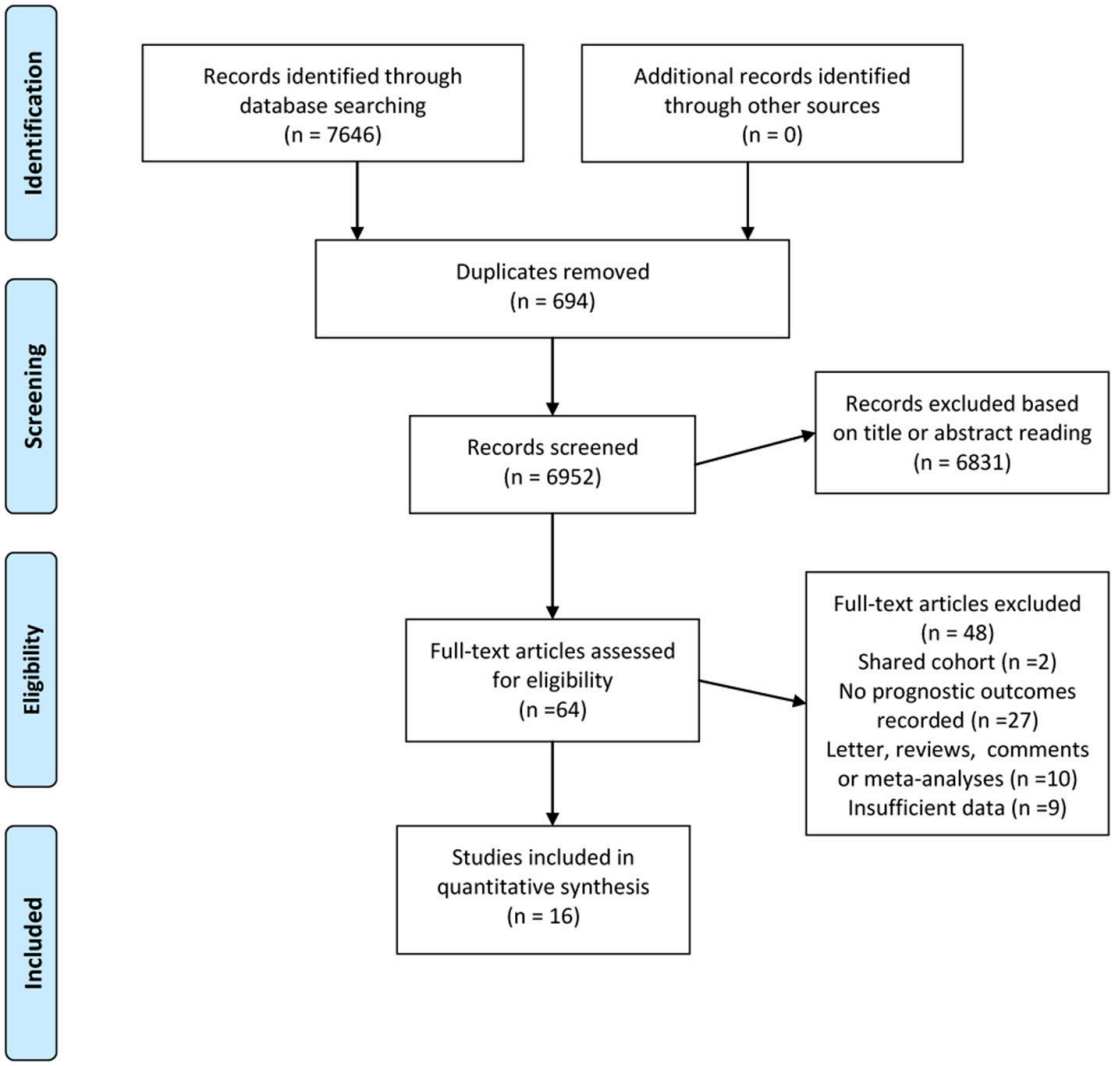
Flow chart of the evidence search and selection process.

Figure 1 demonstrates the detailed process for literature selection and inclusion in this meta-analysis. The searches returned 7,646 records. After excluding 694 duplicates, two authors separately reviewed titles and abstracts and excluded 6,831 irrelevant citations. The remaining 121 relevant citations were then examined for full text review. Finally, 16 studies met the inclusion criteria and were included in this meta-analysis.

### Study characteristics

These 16 cohort studies including 236,022 participants, published between 1995 and 2016 were identified for analysis on the associations between NSAID use and risk of AD (Breitner et al., 1995, 2009; Stewart et al., 1997; in t' Veld et al., 2001; Zandi et al., 2002; Landi et al., 2003; Nilsson et al., 2003; Cornelius et al., 2004; Arvanitakis et al., 2008; Fischer et al., 2008; Szekely et al., 2008; Ancelin et al., 2012; Côté et al., 2012; Chang et al., 2016a,b; Wichmann et al., 2016). Characteristics of the included studies are presented in the Table 1. Eight of the studies examined populations from North America, six studies were from European countries and two studies investigated Asian populations. Twelve of the studies adhered to a prospective cohort design, and four studies were retrospective cohort studies. Sample size ranged from 205 to 166,145. Ten studies investigated the association between aspirin use and risk and AD and three studies examined acetaminophen. In terms of data collection method, six studies collected data by medical records, five by self-reports and five by medical records combined with self-reports.

**Table 1:** Characteristics of the included studies investigating the associations between NSAID exposure and risk of Alzheimer's disease.

**First author**	**Publication year**	**Study design**	**Population origin**	**Sample size**	**Country of origin**	**Race/Ethnicity**	**NSAID type**	**NSAID duration**	**NSAID exposure ascertainment**	**AD ascertainment**	**Risk estimates**	**Follow-up period (years)**	**Adjusted variables**
Wichmann	2016	Prospective cohort	Population-based	4,659	USA	White	Aspirin	Twice/week for more than 3 months	Medical records	Self- or proxy-reported diagnosis	HR	Up to 10 years	Age, sex, and education, smoking, ever heavy drinking, exercise, hypertension, diabetes, myocardial infarction, stroke, angina, statin use, arthritis, self-rated health, body mass index, SF-36 MCS.
Kuang-Hsi Chang	2016	Retrospective cohort	Population-Based	166,145	Taiwan	Asian	NSAID	≤730 days />2191 days	NR	Study clinical investigators	HR	Up to 10 years	Sex, age, and comorbidities of diabetes, hypertension, hyperlipidemia, coronary artery disease, head injury, stroke, COPD, congestive heart failure, and depression
Cheng-Wei Chang	2016	Retrospective Cohort	Population-Based	28,321	Taiwan	Asian	Aspirin	NR	Medical records	Study clinical investigators	HR	11 years	Age group, gender, CCI group, stroke types, anti-diabetic drugs, statins, and hypertensive drugs
Cote	2012	Prospective cohort	Population-based	5,276	Canada	White	NSAID	NR	NR	Self-reported diagnosis	HR	Up to 15 years	Gender, education, adjusted additionally for smoking, alcohol, antioxidant vitamin use, physical activity, arthritis, migraines, comorbidity, and vascular risk factors
Ancelin	2012	Prospective cohort	Community-based	7,234	France	French ethnicity	NSAID	More than once a week	Medical records	Study clinical investigators	HR	7 years	Centre, age, educational level, and baseline cognitive performance, depression, diabetes, hypercholesterolemia, caffeine, smoking, APOE status, ischemic pathologies, chronic joint or back pain, bronchitis, asthma and other chronic respiratory disorders
Breitner	2009	Prospective cohort	Community-based	2,736	USA	Caucasian	NSAID	NR	Medical records and self-report	Study clinical investigators	HR	12 years	Age, sex, APOE status, and education, hypertension, diabetes, body mass index, osteoarthritis, and regular exercise
Szekely	2008	Prospective cohort	Population-Based	3,229	USA	White/ African American	NSAID/aspirin/acetaminophen	NR	Medical records	Study clinical investigators	HR	NR	Age, sex, education level, presence of APOE 4, race (white or African American), and baseline 3MSE
Fischer	2008	Prospective cohort	Community-based	585	Austria	NR	NSAID	NR	Medical records	Study clinical investigators	OR	Up to 2 years	NR
Arvanitakis	2008	Prospective cohort	Population-based	1,019	USA	White	NSAID /aspirin	NR	NR	Study clinical investigators	HR	Up to 12 years	Age, sex, and education
Cornelius	2004	Retrospective cohort	Population-based	1,301	Sweden	Swedish ethnicity	NSAID /aspirin	NR	Medical records	Study clinical investigators	RR	up to 6 years	Age, gender, education
Nilsson	2003	Prospective cohort	Population-based	702	Sweden	Swedish ethnicity	Aspirin	NR	Medical records	Study clinical investigators	RR	9 years	Age, gender, cardiovascular, cerebrovascular diseases
Landi	2003	Prospective cohort	Population-based	2,708	Italy	Caucasian	NSAID/aspirin	NR	Medical records	Study clinical investigators	OR	NR	Age, gender, education, ADL score, CPS score, cerebrovascular diseases, congestive heart failure, hypertension, arrhythmia, depression, PD, diabetes mellitus, thyroid diseases, chronic lung diseases, and concomitant use of antidepressants, antipsychotics, opioid analgesics, and benzodiazepines
Zandi	2002	Prospective cohort	Population-based	3,227	USA	White	NSAID/aspirin	Daily or at least four doses weekly for a month or longer	Medical records	Study clinical investigators	HR	NR	Age, the squared deviation of age from the sample's median value, sex, years of education, the presence of one or two APOE 4 alleles, as well as interactions between age and the APOE 4 terms
in t' Veld	2001	Prospective cohort	Population-based	6,989	Netherlands	Dutch Caucasian	NSAID	< 300 mg per day	Medical records	Study clinical investigators	HR	Mean 6.8 years	Age, sex, level of education, smoking status, and use or non-use of histamine H2-receptor antagonists, hypoglycemic medications, antihypertensive agents, and either oral salicylates or NSAIDs
Stewart	1997	Prospective cohort	Population-based	1,686	USA	White	NSAID/aspirin/acetaminophen	NR	Medical records	Study clinical investigators	RR	15 years	NR
Breitner	1995	Retrospective cohort	Population-based	205	USA	White	NSAID/aspirin/acetaminophen	At least one dose on 4 or more days a week for >1 month/repeated dosage on a schedule for >3 months	Medical records and self-report	Study clinical investigators	HR	NR	Age

*AD, Alzheimer's disease; ADL, Activities of Daily Living scale; APOE, apolipoprotein E; CCI, Charlson-Deyo comorbidity index; COPD, chronic obstructive pulmonary disease; HR, hazard ratio; 3MSE,Modified Mini-Mental State Examination; NR, not reported; NSAID, non-steroidal anti-inflammatory drug; OR, odd ratio; PD, Parkinson disease; RR, relative risk; SF-36 MCS, Short Form Health Survey Mental Component Score*.

### Effect of NSAIDs on the risk of AD based on NSAID type (aspirin and acetaminophen)

**Figure 2:**
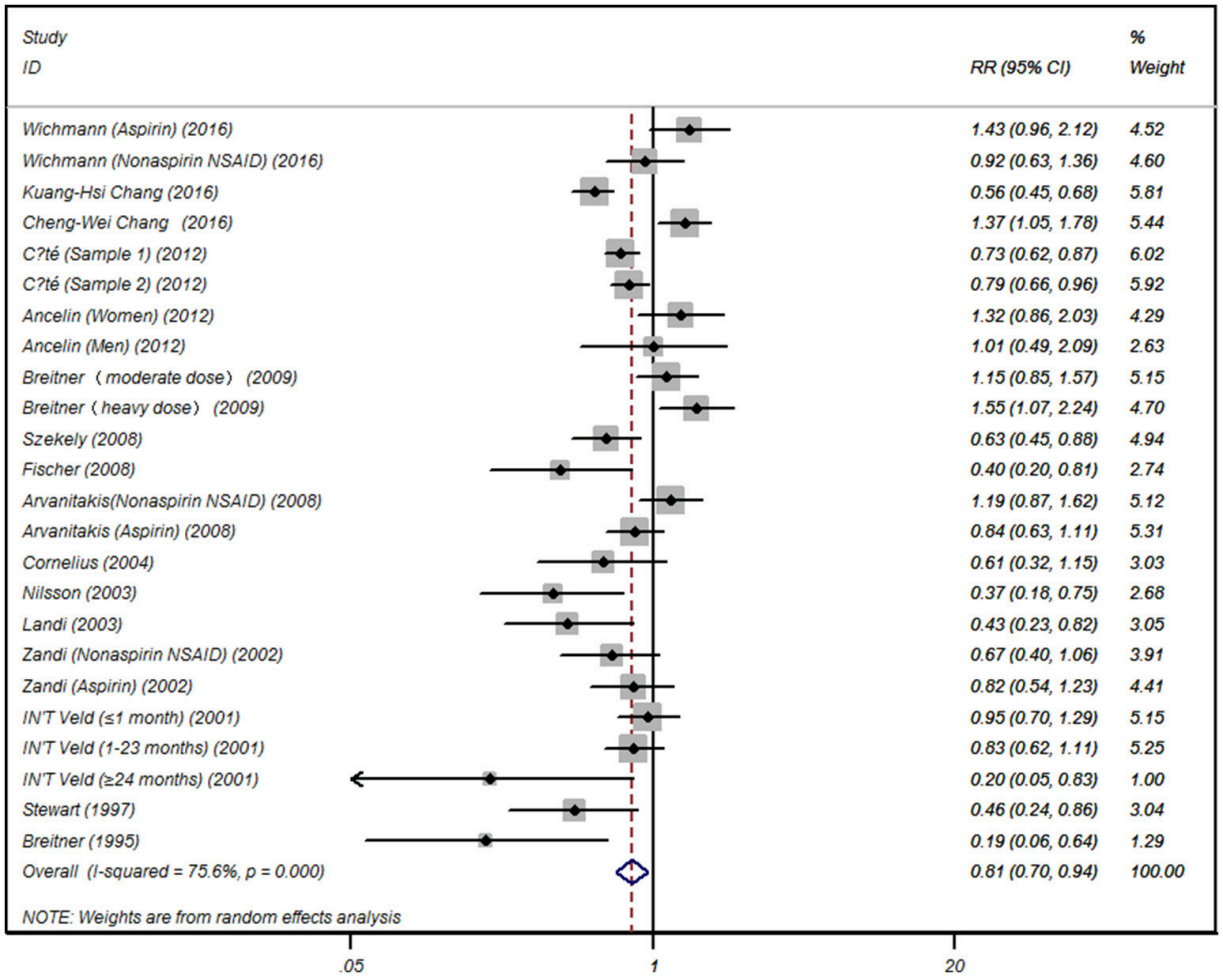
Forest plot for association between NSAID exposure and risk of Alzheimer's disease.

Summarized data from the included 16 studies showed that for any NSAIDs without data separated for aspirin or acetaminophen, NSAID exposure was significantly associated with decreased risk of AD (RR 0.81, 95% CI, 0.70 to 0.94) with significant heterogeneity between studies (*I*^2^ = 75.6, *P* < 0.001) (Figure 2). When the data limited to single type of NSAID, we did not find significant associations between aspirin (RR 0.89, 95% CI, 0.70 to 1.13) or acetaminophen exposure (RR 0.87, 95% CI, 0.40 to 1.91) and risk of AD. Significant association was also not observed for NSAIDs not aspirin exposure (RR 0.84, 95% CI, 0.58 to 1.23) (Table 2).

**Table 2:** Subgroup analyses according to study features.

**Factors**	**RR**	**95% CI**	**Degree of heterogeneity (I^2^ statistics; %)**	**No. of included Studies**	***P* for interaction**
Total	0.81	0.70 to 0.94	75.6	16	NA
Study design					0.186
Prospectively	0.84	0.72 to 0.97	68	12	
Retrospectively	0.64	0.33 to 1.22	90.9	4	
Study setting					<0.001
Population-based	0.76	0.65 to 0.89	73.6	13	
Community-based	1.07	0.75 to 1.53	65.9	3	
Study region					0.561
North America	0.87	0.72 to 1.06	74	8	
Europe	0.72	0.56 to 0.92	61.5	6	
Asia	0.87	0.36 to 2.10	96.3	2	
NSAID type					0.003
Aspirin	0.89	0.70 to 1.13	71	10	
Acetaminophen	0.87	0.40 to 1.91	73.1	3	
All NSAIDs	0.75	0.62 to 0.90	74.3	4	
NSAID not aspirin	0.84	0.58 to 1.23	60.5	11	
Sample size					0.045
≥10000	0.8	0.65 to 0.99	71.8	12	
<10000	0.8	0.64 to 1.01	82	4	
Data collection method				<0.001	
Medical records	0.68	0.50 to 0.93	82.9	6	
Self-reports	0.78	0.67 to 0.91	33	5	
Medical records and self-report	0.97	0.73 to 1.28	73.4	5	

*CI, confidence interval; NSAID, nonsteroidal anti-inflammatory drug; RR, relative risk*.

### Effect of NSAIDs on the risk of AD based on different study settings and study designs

We also categorized the 16 studies into population-based studies (defined as studies recruiting subjects from general population) (*n* = 13) and community-based studies (defined as studies recruiting subjects from community or subgroups of general population) (*n* = 3). NSAID exposure was associated with a reduced risk of AD in the population-based studies (RR 0.76, 95% CI 0.65 to 0.89), but not in the community-based studies (RR 1.07, 95% CI 0.75 to 1.53). Similarly, in the subgroup analysis based on study design, a significant association between NSAID exposure and reduced risk of AD was observed in 12 prospective cohort studies (RR 0.84, 95% CI 0.72 to 0.97), but not in four retrospective cohort studies (RR 0.64,95% CI 0.33 to 1.22) (Table 2).

### Effect of NSAIDs on the risk of AD based on geographical regions

The significant effect of NSAID exposure was exhibited in studies conducted in Europe (RR 0.72, 95% CI 0.56 to 0.92). However, boundary significant effect of NSAID exposure was observed in studies conducted in North America (RR 0.87, 95% CI 0.72 to 1.06). Furthermore, NSAID exposure was not associated with a reduced risk of AD in studies conducted in Asia (RR 0.87, 95% CI 0.36 to 2.10) (Table 2).

### Effect of NSAIDs on the risk of AD based on other factors

We also noted significant effect of NSAID exposure in studies with larger sample size more than 10,000 (RR 0.80, 95% CI 0.65 to 0.99). Moreover, this significant effect was also observed in studies with data collection methods by both medical records (RR 0.68, 95% CI 0.50 to 0.93) and self-reports (RR 0.78, 95% CI 0.67 to 0.91).

### Publication bias

**Figure 3:**
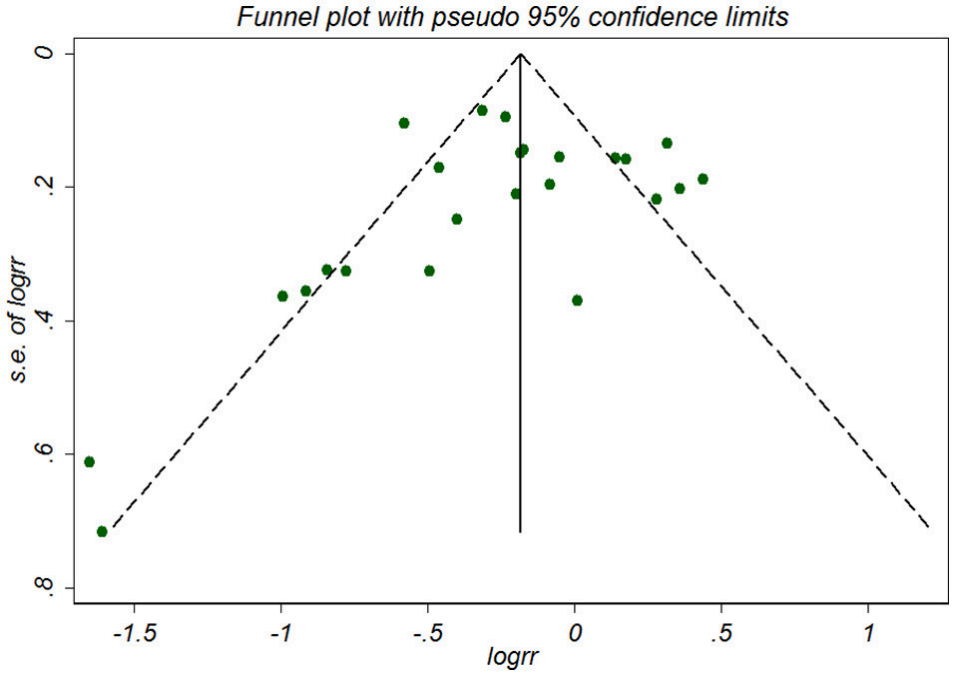
Funnel plot for association between NSAID exposure and risk of Alzheimer's disease. RR, relative risk; logrr; the logarithm of relative risk; s.e. of logrr, standard error of logrr.

No publication bias was detected among the 16 studies according to the Begg's rank correlation test (*P* = 0.07) (Figure 3) and Egger's regression test (*P* = 0.405), respectively. A Duval's non-parametric trim-and-fill method indicated no missing studies for the adjusted estimate, further suggesting the absence of publication bias.

### Discussion

This updated systematic review and meta-analysis of observational studies synthesized the current evidence on the association of NSAID exposure with risk of AD. Overall, synthesis of 16 independent studies identified a moderate (almost 20% risk reduction) protective effect of NSAID use against AD. The findings remained consistent especially for large sample size prospective studies.

Significant between-study heterogeneity was detected in terms of the differences in the investigated populations, NSAID used (type, dose or duration), study design and setting. Actually, the inconsistent findings were to some extent explained by all of the subgroup analyses including the study design and setting, study region, NSAID type, sample size and data collection method.

To the best of our knowledge, the meta-analysis is the most comprehensive one regarding this topic. In 2003, Etminan et al. (Etminan et al., 2003) conducted a meta-analysis of nine observational studies investigating the effect of NSAIDs on risk of AD. They concluded a significant reduction in the risk of AD, but not of aspirin; however, limited studies were involved. Since then, another meta-analysis has reported this topic with inconsistent results. In 2015, Wang et al. (Wang et al., 2015) summarized the data from 12 cohort studies. Though the findings of this meta-analysis were similar to those by Wang et al., which showed that NSAID exposure was significantly associated with reduced risk of AD. However, we did not find this association in individual NSAID type, such as in aspirin or non-aspirin NSAID. Furthermore, we did not investigate the duration response effect for associations between NSAID exposure and AD risk due to limited number of studies and huge between-study heterogeneity, making it meaningless to interpret these results.

Compared to these studies, the present meta-analysis provides more comprehensive evidence, includes a relatively larger number of studies (*n* = 16), establishes subgroup analysis results not previously reported (neutral effect for aspirin, acetaminophen or NSAID not aspirin, significant reduction in AD risk in population-based, prospectively, large sample size studies), updates evidence and provides the direction for future study. Moreover, unlike the previous ones, our meta-analysis did not include case-control studies for analysis as they were less adept at showing a causal relationship than cohort studies. Besides, case-control studies were more prone to bias, especially recall bias. Furthermore, we performed a rigorous and extensive literature search to retrieve all eligible studies according to Cochrane handbook including PubMed, Embase and Cochrane Library, which was considered as a standard search for systematic reviews.

However, several limitations have to be addressed in interpreting the results of this meta-analysis. Firstly, significant differences were noted in terms of study design or setting, and type of NSAID exposure. Though sources of heterogeneity were investigated through detailed subgroup analyses, the summary risk estimates are still based on heterogeneous data which had to be interpreted cautiously. Secondly, different studies used different adjusted variables controlled for confounders; however, some unknown or unmeasured variables that might explain the heterogeneity could not be fully adjusted. Finally, unpublished studies were not searched and we did not contact authors for missing original data. Though visual inspection of the funnel plot and Egger's regression and Begg's rank correlation tests did not indicate suspicion of small-study effects, we should still interpret the results with caution.

In conclusion, current evidence suggests that NSAID exposure might be significantly associated with reduced risk of AD, especially for large prospective population-based cohort studies, in contrast, we found no such evidence for aspirin, acetaminophen or NSAID not aspirin. However, as the strength of the associations was really weak, we have to interpret it with caution. Therefore, further larger prospective study is warranted to confirm or refute these findings. Moreover, more critical issues, such as the effects of the dose and duration of NSAID exposure should be further investigated.

## Author contributions

CZ: Conception and design; CZ and YW: Collection and assembly of data; CZ, YW, DW, JZ, and FZ: Data analysis and interpretation; CZ: Manuscript writing; CZ, YW, DW, JZ, and FZ: Final approval of manuscript.

### Conflict of interest statement

The authors declare that the research was conducted in the absence of any commercial or financial relationships that could be construed as a potential conflict of interest.
