# Supplementary material for: NSAID Exposure and Risk of Alzheimer's Disease: An Updated Meta-Analysis From Cohort Studies
Source: Front Aging Neurosci. 2018 Mar 28;10:83. doi: 10.3389/fnagi.2018.00083 (PMC5882872; doi:10.3389/fnagi.2018.00083)
Supplement: Supplementary file 1 [file Table1.DOCX]

**Supplementary Search Strategy**

Search data: April 15 2017

**Table A1. Pubmed**

| 1. " Alzheimer Disease "[Mesh] |
| --- |
| 1. (Alzheimer* [Title/Abstract]) OR dement* [Title/Abstract] |
| 1. 1 OR 2 |
| 1. " Anti-Inflammatory Agents, Non-Steroidal"[Mesh] |
| 1. (NSAIDs) [Title/Abstract] OR (NSAID) [Title/Abstract] |
| 1. (non-steroidal anti-inflammatory drug*) [Title/Abstract] |
| 1. (ampyrone OR antipyrine OR apazone OR aspirin OR bufexamac OR clofazimine OR clonixin OR curcumin OR dapsone OR diclofenac OR diflunisal OR dipyrone OR epirizole OR etodolac OR fenoprofen OR flurbiprofen OR glycyrrhizic acid OR ibuprofen OR indomethacin OR ketoprofen OR ketorolac OR ketorolac tromethamine OR meclofenamic acid OR mefenamic acid OR mesalamine) [Title/Abstract] |
| 1. 4 OR 5 OR 6 OR 7 |
| 1. 3 AND 8 |

**Table A2. Embase**

| 1. 'alzheimer disease'/exp |
| --- |
| 1. (Alzheimer* OR dement*):ab,ti |
| 1. 1 OR 2 |
| 1. 'nonsteroid antiinflammatory agent'/exp |
| 1. (NSAIDs OR NSAID OR ampyrone OR antipyrine OR apazone OR aspirin OR bufexamac OR clofazimine OR clonixin OR curcumin OR dapsone OR diclofenac OR diflunisal OR dipyrone OR epirizole OR etodolac OR fenoprofen OR flurbiprofen OR ‘glycyrrhizic acid’ OR ibuprofen OR indomethacin OR ketoprofen OR ketorolac OR ‘ketorolac tromethamine’ OR ‘meclofenamic acid’ OR mefenamic acid OR mesalamine):ab,ti |
| 1. 4 OR 5 |
| 1. 3 AND 6 |

**Table A3. Cochrane Library**

| 1. MeSH descriptor: [Alzheimer Disease] explode all trees |
| --- |
| 1. (Alzheimer* or dement*):ti,ab,kw (Word variations have been searched) |
| 1. 1 OR 2 |
| 1. MeSH descriptor: [Anti-Inflammatory Agents, Non-Steroidal] explode all trees |
| 1. (NSAIDs or NSAID or ampyrone or antipyrine or apazone or aspirin or bufexamac or clofazimine or clonixin or curcumin or dapsone or diclofenac or diflunisal or dipyrone or epirizole or etodolac or fenoprofen or flurbiprofen or ‘glycyrrhizic acid’ or ibuprofen or indomethacin or ketoprofen or ketorolac or ‘ketorolac tromethamine’ or ‘meclofenamic acid’ or mefenamic acid or mesalamine):ti,ab,kw (Word variations have been searched) |
| 1. 4 OR 5 |
| 1. 3 AND 6 |

**Supplementary Table S1.** Selection procedure of included and excluded studies

| **Study** | **Year** | **Design** | **Selection** | **Comparability** | **Outcome/exposure** | **Overall quality (max 9)** |
| --- | --- | --- | --- | --- | --- | --- |
| Wichmann | 2016 | cohort | 4 | 2 | 2 | 8 |
| Kuang-Hsi Chang | 2016 | cohort | 3 | 2 | 2 | 7 |
| Cheng-Wei Chang | 2016 | cohort | 4 | 2 | 2 | 8 |
| Cote | 2012 | cohort | 3 | 2 | 3 | 8 |
| Ancelin | 2012 | cohort | 4 | 2 | 3 | 9 |
| Breitner | 2009 | cohort | 3 | 2 | 3 | 8 |
| Szekely | 2008 | cohort | 4 | 2 | 1 | 7 |
| Fischer | 2008 | cohort | 4 | 1 | 3 | 8 |
| Arvanitakis | 2008 | cohort | 3 | 2 | 3 | 8 |
| Cornelius | 2004 | cohort | 4 | 2 | 2 | 8 |
| Nilsson | 2003 | cohort | 4 | 1 | 2 | 7 |
| Landi | 2003 | cohort | 4 | 2 | 1 | 7 |
| Zandi | 2002 | cohort | 4 | 2 | 1 | 7 |
| IN'T Veld | 2001 | cohort | 4 | 2 | 2 | 8 |
| Stewart | 1997 | cohort | 4 | 1 | 2 | 7 |
| Breitner | 1995 | cohort | 3 | 2 | 1 | 6 |

Note: Study quality assessment of observational studies performed using the Newcastle–Ottawa scale.
